# Pulmonary Embolism Following Quadriceps Tendon Repair With Tourniquet Use Despite Adequate Thromboprophylaxis: A Case Report

**DOI:** 10.7759/cureus.94718

**Published:** 2025-10-16

**Authors:** Muthusankar Sudalaimuthu, Bharath Sundaramoorthy, Suresh Kumar Gopala Pillai

**Affiliations:** 1 Emergency Medicine, Swansea Bay University Health Board, Swansea, GBR; 2 General Medicine, Government Thoothukudi Medical College, Thoothukudi, IND; 3 Emergency Medicine, Morriston Hospital, Swansea, GBR

**Keywords:** anticoagulation, morbid obesity, orthopedic surgery, pulmonary embolism, quadriceps tendon repair, thromboprophylaxis, tourniquet use, venous thromboembolism

## Abstract

Venous thromboembolism (VTE) is a recognized complication following orthopaedic surgery of the lower limb, but its incidence after quadriceps tendon repair remains poorly described. We report the case of a 53-year-old man (BMI 42 kg/m²) with morbid obesity who developed acute pulmonary embolism (PE) following quadriceps tendon repair performed with tourniquet assistance, despite receiving extended perioperative thromboprophylaxis with tinzaparin. This case highlights the interplay between tourniquet use, patient-specific risk factors (obesity, delayed surgery, and reduced mobility), and the adequacy of standard-duration prophylaxis. We also discuss why existing arthroplasty data may not directly apply to tendon repair surgery and the need for individualized prophylaxis strategies in high-risk patients.

## Introduction

Venous thromboembolism (VTE) is a potentially life-threatening complication after lower limb orthopaedic procedures. While its incidence and prevention strategies are well described in total hip and knee arthroplasty, data specifically addressing VTE risk following quadriceps tendon repair are sparse, with only a handful of case reports and small observational studies available [1,2,3]. Risk factors such as obesity, immobility, and delayed surgical intervention further increase susceptibility. The use of pneumatic tourniquets remains common in lower limb surgery, as they facilitate visualization and reduce intraoperative blood loss. However, tourniquet use can induce venous stasis and endothelial injury, potentially increasing thromboembolic risk. Large arthroplasty cohorts have reported no significant association between tourniquet use and VTE [4], but these findings may not be generalizable to tendon repair populations.

We present the case of a 53-year-old man with morbid obesity who developed acute pulmonary embolism (PE) after quadriceps tendon repair performed with tourniquet assistance, despite extended perioperative thromboprophylaxis. This report underscores the potential association between tourniquet use, cumulative risk factors, and the adequacy of standard prophylactic regimens.

## Case presentation

A 53-year-old man (BMI 42 kg/m²) with morbid obesity and a history of recurrent falls presented to the fracture clinic with suspected quadriceps tendon rupture, confirmed on ultrasound. There was no personal or family history of venous thromboembolism or known hereditary thrombophilia.

Surgery was delayed for two weeks due to a superficial peri-knee lesion requiring local care. During this waiting period, he received prophylactic tinzaparin 4,500 IU subcutaneously once daily for three weeks.

He subsequently underwent right quadriceps tendon repair under general anaesthesia with a femoral nerve block. A pneumatic tourniquet was inflated on the right thigh during the procedure (duration: 65 minutes). Operative findings confirmed complete rupture of the quadriceps tendon, which was repaired using four rows of Ethion sutures in a Krakow fashion, secured through three osseous patellar tunnels with an ACL guidewire. The repair was reinforced with Vicryl and a triple-folded mesh, followed by thorough washout and layered closure. Postoperatively, the knee was immobilized in full extension.

He mobilized with physiotherapy using crutches and received tinzaparin 4,500 IU once daily for one week postoperatively before discontinuation. Four days after stopping prophylaxis, he developed right calf discomfort, which resolved spontaneously. Shortly thereafter, he experienced acute left basal pleuritic chest pain radiating posteriorly, associated with progressive dyspnoea but no cough, haemoptysis, or trauma. Wells' score was 9.

**Figure 1:**
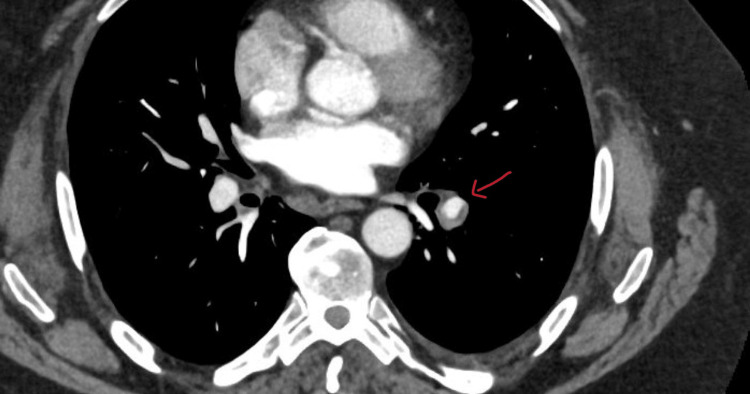
CT pulmonary angiography demonstrating an acute pulmonary embolism at the bifurcation of the left lower lobe pulmonary artery

CT pulmonary angiogram demonstrated acute PE at the bifurcation of the left lower lobe pulmonary artery, extending into segmental branches (Figure 1). There was no right heart strain or reflux into the inferior vena cava. Duplex ultrasound of the lower limbs was not performed. He was commenced on therapeutic anticoagulation (apixaban) with good clinical recovery. A thrombophilia screen was negative.

## Discussion

This case demonstrates the occurrence of PE following quadriceps tendon repair despite extended perioperative thromboprophylaxis, suggesting that standard-duration regimens may be insufficient in certain high-risk patients. The development of PE soon after stopping tinzaparin implies that the thrombotic risk persisted beyond the prophylaxis window, highlighting the importance of individualized thromboprophylaxis strategies in orthopedic cases outside the arthroplasty setting.

Several interrelated factors likely contributed to thrombus formation in this patient, including morbid obesity (BMI 42 kg/m²), postoperative immobility, delayed surgery, and intraoperative tourniquet use. Morbid obesity is well recognised to promote venous stasis and hypercoagulability, both central to Virchow’s triad. In addition, knee immobilisation in full extension markedly reduces calf muscle pump function, further predisposing to venous stasis. The two-week preoperative delay extended this period of reduced mobility, compounding risk before surgery.

Tourniquet use may also have played an important contributory role. Although pneumatic tourniquets are routinely applied to optimise visualisation and limit blood loss, their application produces transient venous occlusion and potential endothelial injury. Both mechanisms facilitate local thrombus formation. Several case reports have documented deep vein thrombosis and PE following tendon repair procedures in which tourniquets were used, even when standard prophylaxis was given [1,2,5].

By contrast, large arthroplasty studies such as the Danish multicentre cohort of over 16,000 total knee arthroplasties reported no increased risk of postoperative VTE with tourniquet use [4]. However, extrapolating these findings to tendon repair is problematic. Tendon repair patients often differ markedly from arthroplasty cohorts in their baseline comorbidities, tourniquet duration, and rehabilitation protocols. Unlike arthroplasty patients, those undergoing tendon repair may experience prolonged immobilisation, slower recovery, and less standardised perioperative care. Consequently, arthroplasty data may underestimate the thromboembolic risk in tendon repair populations.

The 2022 CHEST guidelines emphasize that thromboprophylaxis should be tailored to individual risk rather than procedure type alone [6]. In light of this case, clinicians should consider extending pharmacological prophylaxis to at least two to four weeks postoperatively, or until the patient achieves full mobilisation, especially when multiple risk factors coexist. Additionally, a critical assessment of the necessity and duration of tourniquet use is warranted in such high-risk individuals. Overall, this case underscores the need for a more personalised and cautious approach to VTE prevention in lower limb tendon repair surgery.

Several reports have described venous thromboembolism following tendon repairs and related lower limb surgeries in which tourniquet use was employed. A summary of these published cases, including procedure type, tourniquet use, thromboprophylaxis strategy, and clinical outcome, is presented in Table 1.

**Table 1:** Reported cases of venous thromboembolism following quadriceps tendon repair and related lower limb surgeries with tourniquet use Table summarizing published reports of venous thromboembolism (VTE) following quadriceps tendon repair and related lower limb surgeries where tourniquet use was applied. Abbreviations: PE, pulmonary embolism; DVT, deep vein thrombosis; LMWH, low-molecular-weight heparin; ACL, anterior cruciate ligament

Author / year	Procedure	Tourniquet use	VTE prophylaxis	VTE event	Key point
Cedeño-Rodriguez et al., 2025 [1]	Bilateral quadriceps tendon repair	Unilateral tourniquet	Guideline-adherent prophylaxis	PE (ipsilateral)	Highlighted paradoxical risk despite prophylaxis; questioned tourniquet contribution.
Hess, 2010 [2]	Quadriceps tendon rupture repair	Yes	LMWH post-op	DVT + PE	Suggested venous stasis from the tourniquet may have precipitated the clot.
Petersen et al., 2019 [3]	Total knee arthroplasty	Yes	LMWH + stockings	Fatal PE	Suggested possible role of tourniquet-related stasis.
Sun et al., 2012 [4]	ACL reconstruction	Yes (60–90 min)	Standard prophylaxis	DVT (calf veins)	Higher DVT rates linked with longer tourniquet duration.
O’Shea et al., 2002 [5]	Quadriceps tendon rupture	Yes	Not specified	Symptomatic PE	Raised awareness of VTE risk after tendon repair.

## Conclusions

This case illustrates a potential association between tourniquet use, patient-specific risk factors, and postoperative PE following quadriceps tendon repair. Despite extended perioperative thromboprophylaxis, VTE developed shortly after cessation, suggesting that standard-duration regimens may be inadequate for patients with morbid obesity, delayed surgery, or restricted mobility. These findings highlight the importance of individualised prophylaxis planning, including consideration of extended anticoagulation and cautious intraoperative tourniquet use in high-risk individuals. Further prospective studies focusing on non-arthroplasty lower limb surgeries are needed to better define these risks and guide the development of more tailored prevention strategies.
